# Stability of SARS-CoV-2 on Produce following a Low-Dose Aerosol Exposure

**DOI:** 10.4269/ajtmh.20-1033

**Published:** 2020-09-14

**Authors:** Andrew D. Haddow, Taylor R. Watt, Holly A. Bloomfield, Joshua D. Shamblin, David N. Dyer, David E. Harbourt

**Affiliations:** 1 General Dynamics Health Solutions in Support of USAMRIID, Fort Detrick, Maryland;; 2 United States Army Medical Research Institute of Infectious Diseases (USAMRIID), Fort Detrick, Maryland

## Abstract

We modeled the stability of SARS-CoV-2 on apples, tomatoes, and jalapeño peppers at two temperatures following a low-dose aerosol exposure designed to simulate an airborne transmission event involving droplet nuclei. Infectious virus was not recovered postexposure.

Transmission of SARS-CoV-2, the causative agent of COVID-19, primarily occurs through respiratory droplets, although increasing evidence suggests the potential for airborne transmission.^1,2^ However, fomites may act as a secondary transmission mode.^3–5^ Before purchase, produce is commonly handled by and exposed to multiple persons, including staff and shoppers, therefore increasing the likelihood of contamination via infectious respiratory droplets (> 5 µm) and/or droplet nuclei (≤ 5 µm). Herein, we carried out a pilot study to model the stability of SARS-CoV-2 on apples, tomatoes, and jalapeño peppers at two temperatures following an aerosol exposure designed to simulate a low-dose SARS-CoV-2 airborne transmission event involving droplet nuclei.

Certified organic produce was obtained from a local grocery store—selections being made at random. After which, produce was surface-disinfected by ultraviolet light for 10 minutes, and then stabilized in perforated metal boxes using modeling clay (base of each piece) to prevent movement during aerosol exposure. Produce was exposed to SARS-CoV-2 (USA-WA1/2020, GenBank accession no. MN985325.1) via a three-jet Collison nebulizer (BGI, Waltham, MA) for 10 minutes. Apples were exposed to 3.1 log_10_ plaque forming unit (PFU)/L of air, whereas tomatoes and jalapeño peppers were exposed to 3.2 log_10_ PFU/L of air. Particle size mass median aerodynamic diameter was 1.35 µm (apples), 1.38 µm (tomatoes), and 1.35 µm (jalapeño peppers), with a geometric SD of 1.82 (apples), 1.91 (tomatoes), and 1.87 (jalapeño peppers).

Samples were collected via swabbing (to mimic touching during produce handling) using the BD Universal Viral Transport System (Becton, Dickinson, and Company, Sparks, MD) from exposed produce stored at two temperatures (22 ± 2°C “room temperature” and 4 ± 2°C “refrigerated temperature”), at four time points postexposure (1, 4, 8, and 24 hours ± 2 minutes). Three samples (triplicate) were taken from each produce type at each time point with a swab pass of approximately 7 cm in length, after which the swab was flipped over to the clean side, and a second pass was made in a different area, again the length of swab pass being approximately 7 cm. The length of the fiber swab was approximately 1.5 cm; thus, the total surface area swabbed from each sample was approximately 21 cm^2^. Consequently, the total sampling area per produce type was approximately 63 cm^2^ at each temperature and time point. Previously swabbed areas were not re-swabbed. Plastic identification stickers, known as price lookup stickers, commonly found on pieces of produce were not swabbed. Samples were stored at −80°C before quantification of infectious virus. Virus titration was performed via plaque assay on Vero 76 cells (ATCC, Manassas, VA; CRL-1587) as previously described,^4^ with the limit of detection being 1.0 log_10_ PFU/mL. We did not detect infectious virus on produce at any time point post-exposure.

The results of this study indicate fomite transmission via produce exposed to a low-dose SARS-CoV-2 aerosol is likely a rare event. However, the results of this study should be interpreted in light of its limitations. Produce was not immediately cooled to 4 ± 2°C post-exposure to permit the transportation of produce from the aerosol chamber and to allow swabbing at 1 hour post-exposure. Thus, it is possible that degradation of viable virus may have occurred during this time period while produce was held at 22 ± 2°C. The small particle size of the aerosol likely exacerbated viable virus degradation due to desiccation. Although we did not swab the plastic identification stickers commonly found on individual pieces of produce, it is possible that infectious virus may be more stable on these impervious surfaces.^3^ While we did not detect any infectious virus post-exposure, we used virus grown in media for this experiment. Infectious virus contained in respiratory droplets, droplet nuclei, and/or mucus during the course of a natural infection might help stabilize the virus for a longer duration. In addition, a higher exposure dose may deposit sufficient virus particles to permit the recovery of infectious virus. Thus, the potential for fomite transmission may still exist when handling produce, and individuals should practice proper hand hygiene and wear masks to reduce SARS-CoV-2 transmission potential. Persons are advised to consult the CDC guidelines for the safe handling and preparation of produce and other food items for update-to-date information during the COVID-19 pandemic.^6,7^

In summary, we demonstrated there is a low potential for infectious SARS-CoV-2 to be detected on apples, tomatoes, and jalapeño peppers at 1 hour following a low-dose experimental aerosol exposure.
